# The Emergence of Group Potency and Its Implications for Team Effectiveness

**DOI:** 10.3389/fpsyg.2019.00992

**Published:** 2019-05-03

**Authors:** Hayden J. R. Woodley, Matthew J. W. McLarnon, Thomas A. O’Neill

**Affiliations:** ^1^Faculty of Business, University of Prince Edward Island, Charlottetown, PE, Canada; ^2^Department of Psychology, Oakland University, Rochester, MI, United States; ^3^Department of Psychology, University of Calgary, Calgary, AB, Canada

**Keywords:** group potency, emergence, team effectiveness, conscientiousness, extraversion

## Abstract

Much of the previous research on the emergence of team-level constructs has overlooked their inherently dynamic nature by relying on static, cross-sectional approaches. Although theoretical arguments regarding emergent states have underscored the importance of considering time, minimal work has examined the dynamics of emergent states. In the present research, we address this limitation by investigating the dynamic nature of group potency, a crucial emergent state, over time. Theory around the “better-than-average” effect (i.e., an individual’s tendency to think he/she is better than the average person) suggests that individuals may have elevated expectations of their group’s early potency, but may decrease over time as team members interact gain a more realistic perspective of their group’s potential. In addition, as members gain experience with each other, they will develop a shared understanding of their team’s attributes. The current study used latent growth and consensus emergence modeling to examine how potency changes over time, and its relation with team effectiveness. Further, in accordance with the input-process-output framework, we investigated how group potency mediated the relations between team-level compositions of conscientiousness and extraversion and team effectiveness. We collected data at three time points throughout an engineering design course from 337 first-year engineering students that comprised 77 project teams. Results indicated that group potency decreased over time in a linear trend, and that group consensus increased over time. We also found that teams’ initial potency was a significant predictor of team effectiveness, but that change in potency was not related to team effectiveness. Finally, we found that the indirect effect linking conscientiousness to effectiveness, through initial potency, was supported. Overall, the current study offers a unique understanding of the emergence of group potency, and facilitate a number theoretical and practical implications, which are discussed.

## Introduction

According to the input-process-outcome (IPO) framework (McGrath, 1964) and related models (e.g., the input-mediator-output-input [IMOI] model; Ilgen et al., 2005), emergent states are integral to understanding the effectiveness of teams. In this light, extensive research has been conducted in effort to improve our understanding of how emergent states influence team effectiveness (Kozlowski and Ilgen, 2006). Marks et al. (2001) defined emergent states as, “constructs that characterize properties of the team that are typically dynamic in nature and vary as a function of team context, inputs, processes, and outcomes” (p. 357). Examples of emergent states include collective efficacy, group potency, and cohesion. Overall, meta-analyses have found that the previously mentioned emergent states are positively related to team effectiveness (e.g., Gully et al., 2002; Beal et al., 2003; Stajkovic et al., 2009, respectively). Although these findings have been influential in building our understanding of team effectiveness, little research has investigated the temporal, dynamic aspects of emergent states (Kozlowski et al., 2016; Waller et al., 2016). Ilgen et al. (2005) argued that time plays an important role in understanding the emergence of states in teams, and without more direct insight into the temporal nature of emergent team processes, theoretical advancements, and practical recommendations will be limited (see also Collins et al., 2016; Salas et al., 2017a,b). To address this issue, the current investigation sought to examine: (1) how group potency, a critical emergent state, changes over time, (2) the relation between the dynamics of potency and team effectiveness, and (3) the mediating effect the dynamics of potency have on the relation between inputs (i.e., team-level personality) and team effectiveness.

In this research, data were gathered from student engineering project teams over multiple time points during an academic course. We then used latent growth and consensus emergence modeling to examine the dynamic nature and emergent properties of group potency. Throughout, we use the term *dynamic* to reflect the separate factors of the initial starting point of teams’ potency, the rate of change in potency over time, and also the emergence of the construct (see Ployhart and Vandenberg, 2010; Wang et al., 2016). Further, we investigated the role of team-level input variables (i.e., team-level conscientiousness and extraversion) as predictors of the dynamicity of group potency. Additionally, we examined whether the dynamics of group potency mediated the relations for both conscientiousness and extraversion on team effectiveness.

In the following sections, we utilize conservation of resources (COR) theory to discuss the importance of group potency as a team-level resource that influences team effectiveness, in accordance within the broad IPO and IMOI frameworks. In addition, we invoke COR to support our theoretical rationale for how potency changes over time, and how this change predicts team effectiveness. Then, we theorize that specific personality traits (i.e., conscientiousness and extraversion) are both antecedents (i.e., inputs) and resources that contribute to the process of group potency dynamics and the prediction of team effectiveness.

## Group Potency

Group potency is one of the most frequently investigated emergent states and team processes associated with effective teamwork (LePine et al., 2008), and recent research suggest this trend is going to continue (e.g., O’Neill et al., 2016; Schaubroeck et al., 2016, among others). Although it has been described in different forms previously (see Stajkovic et al., 2009), we adhere to its conventional definition as a team’s generalized confidence in its ability to perform across a variety of situations (see Guzzo et al., 1993). Potency differs from efficacy, in that “efficacy represents a shared, task-specific expectation that the team can accomplish its goals, whereas potency is a more generalized sense of competence” (Kozlowski, 2018, p. 208). To date, two meta-analyses have investigated the relations between group potency and team performance (Gully et al., 2002; Stajkovic et al., 2009), with both reporting that group potency is positively related to team performance, ρ = 0.35 and 0.29, respectively.

Nevertheless, these meta-analyses are based on research that has used static, cross-sectional approaches (Marks et al., 2001), which unfortunately may not adequately address the inherently dynamic nature of group potency. As such, the dynamic aspects of group potency, which we expand on subsequently, have been relatively ignored by past research (Kozlowski and Ilgen, 2006; cf. Collins and Parker, 2010; Collins et al., 2016; Salas et al., 2017a,b). There are two potential reasons for this: (1) gathering longitudinal data with teams can be difficult because team membership and/or project assignments may change over time (see McClurg et al., 2017), and (2) the analytical approaches for investigating emergence and growth had not developed until recently (see Collins et al., 2016; Lang et al., 2018). In this research, we address these methodological challenges and present a novel investigation into the dynamics of group potency over time.

## Emergence

The concept of emergence in multilevel phenomena (e.g., teams) has been the focus of recent theoretical discussions (see Kozlowski et al., 2013; Waller et al., 2016; Grossman et al., 2017). Here, we establish a theoretical model for the emergence and dynamics involved with group potency. Kozlowski and Klein (2000) defined an emergent state as a characteristic of a team that “is amplified by their interactions, and manifested as a higher-level, collective phenomenon” (p. 55). An emergent state, therefore, is a dynamic construct, which theoretically changes or emerges over time (Kozlowski and Ilgen, 2006). We adopt this as the basis for our investigation because it makes an important distinction that other definitions do not address (e.g., Marks et al., 2001). In Kozlowski and Klein (2000) definition, emergence is not a singular attribute; rather there are two distinct underlying processes that develop as a result of group interactions: (1) amplification, and (2) consensus. Amplification refers to the growth aspect, or in broader terms, reflects the notion of changing levels over time, of a construct. Consensus refers to the emergence of a collective phenomenon from the shared perceptions of individual members. Broadly speaking, the literature on emergent states has ignored the dynamic nature of both amplification and consensus (Cronin et al., 2011; Kozlowski et al., 2016). In particular, the vast majority of previous research has used cross-sectional data, which is poorly suited to examining the role time plays in both amplification and consensus processes (Cronin et al., 2011; Roe et al., 2012; Vantilborgh et al., 2018). Emergent states should demonstrate changes in level and consensus over time, and result from team interactions and collective experiences that lead to increasingly shared perceptions and consensus between individual members (Kozlowski and Klein, 2000; Marks et al., 2001; Kozlowski et al., 2013; Kozlowski, 2018).

### Group Potency Levels Across Time

For group potency – and other emergent states – to develop, team members need time and a reason to interact and develop an understanding of “who they are” as a group (Marks et al., 2001; Kozlowski, 2018). This suggests that potentially, at first, teams would be less confident in their ability to perform because they do not have enough experience with each other to develop a shared understanding of their collective ability. Then, conceivably, as team members interact over time they will gain insight into each member’s work habits and abilities, leading to increases in collective confidence. This perspective, however, rests on the assumption that team members enter teams without any pre-existing expectations. It seems more likely that team members enter their teams with high expectations, optimism, and confidence, especially without evidence to suggest otherwise. In support of the latter, Allen and O’Neill (2015b) theorized that the early agreement they found among team members on ratings of emergent states (e.g., group potency) might be attributed to an early positivity bias. They reasoned that this bias may lead to inflated perceptions of potency early in teams’ lifecycle, indicating a strong need to consider the role of time in investigating team processes. Unfortunately, limited research has been conducted on the dynamic nature of group potency. One study, however, by Lester et al. (2002) measured group potency at two time points, and using differences scores found that group potency *decreased* over time. Although difference scores have several methodological shortcomings (see Edwards, 2001, for a review), this finding is not overly surprising. In fact, research on the “better-than-average” effect (e.g., Svenson, 1981) – a common social comparison bias – would suggest that team members’ initial expectations of their team’s collective general ability might be inflated. The better-than-average effect has also been found to be stronger when the comparison target is ambiguous (Alicke et al., 1995), as in a newly formed team might be, and is positively related to over-confidence in one’s individual ability (Larrick et al., 2007). It may therefore stand to reason that confidence in one’s team may occur early in a team’s lifecycle. Yet, as members may rate their team artificially high early on in their tenure (Lester et al., 2002), scores will tend to decrease over time as members interact with each other and face ongoing challenges with the task that may reduce their potency resources that are available for subsequent performance episodes. Continuing interactions and experience with the task may facilitate more realistic perceptions of how the team can reasonably be expected to perform (i.e., a demonstrating a decreasing trend over time), in conjunction with increasing consensus across members. Together, this underscores the emergent and dynamic nature of potency. Based on this theorizing, the following is hypothesized:

Hypothesis 1: Perceptions of group potency will decrease over time.

To be clear, we suggest that the downward trend of group potency would be approximated well by a linear trajectory (see Ployhart and Vandenberg, 2010). Rather than a series of discrete step-wise drops, or patterns of punctuated change, we anticipate an incremental series of changes over time. Particularly, as teams meet on a set schedule during their lifecycle (i.e., three times a week during course and laboratory sessions) interacting with each other may lead to gradual changes in perceptions of group potency. Thus, rather than sudden, dramatic changes (i.e., discontinuous, non-linear change) in perceptions of group potency, teams will demonstrate a consistent, linear, downward pattern over time.

#### Group Potency Over Time and Implications for Team Effectiveness

To improve our understanding of the dynamic nature of group potency, it is crucial to investigate its criterion-related validity and examine how group potency relates to team effectiveness. Meta-analytic research at both the individual- (e.g., Stajkovic and Luthans, 1998) and team-level (Gully et al., 2002; Stajkovic et al., 2009) suggests strong, positive relations with performance. However, these results, as previously mentioned, are based on static research methods and do not take into consideration changes over time.

Within a time-limited project, group potency may function as a team-level resource that takes time to coalesce through consensus, but can be drawn upon by the team to influence effectiveness and the achievement of team tasks and goals. According to the COR theory, resources play an important role in understanding behavioral outcomes (e.g., performance; Halbesleben and Bowler, 2007). Halbesleben et al. (2014) defined resources as “anything perceived by the individual to help attain his or her goal” (p. 1338). Although defined at the individual level, this definition could easily be translated to the team context by defining a team resource as anything perceived by the members that can help the team attain its goal(s). This definition allows group potency to be considered a team-level resource that can be used to optimally influence team effectiveness (see Guzzo et al., 1993; Gully et al., 2002; Stajkovic et al., 2009). In this light, there are two key components of COR to consider: (1) initial resource losses lead to future resource losses, and (2) a greater amount of a resource can reduce the vulnerability to resource losses (Hobfoll, 2001, 2011), as in a buffering effect. Concerning initial resource loss, Hobfoll et al. (2018) argued that resource loss begets stress, which leads to further resource loss. In support of this theorizing, research by Demerouti et al. (2004) demonstrated that resource loss (due to work pressure) leads to increased stress (i.e., work-life role conflict) in individuals, which then leads to further resource loss (i.e., exhaustion). Demerouti et al. (2004) referred to this phenomenon as a “loss spiral,” which has also been reported by De Cuyper et al. (2012) and Whitman et al. (2014). Consistent with these findings, we anticipate that teams that are unable to conserve their potency resources over time will lose further resources over time, and experience worse team effectiveness. Concerning the buffering effect, Hobfoll et al. (2018) argued that individuals who have more resources are less likely to lose resources and are more likely to gain resources. For example, Hakanen et al. (2008) found that individuals with greater job resources were more engaged in their work, which led to increased innovativeness in their work group. Chen et al. (2009) also found that by boosting individuals’ resources through training, they were more likely to adapt to changing work contexts and were less likely to experience resource loss (i.e., exhaustion). We therefore propose that teams that start with higher potency (i.e., initially have more potency resources than other teams) will perform better than teams that have lower initial potency. Together, we therefore hypothesize the following:

*Hypothesis 2: Changes in group potency* (i.e., *the downward trend described by Hypothesis 1*) *will be negatively related to team effectiveness.*
Hypothesis 3: Initial group potency will be positively related to team effectiveness.

### Group Potency Consensus Over Time

Emergent states, as previously defined, describe the development of a collective phenomenon from the sharedness of individual members’ perceptions of a team-level attribute. Emergent states therefore exist as constructs at the collective level (e.g., team, group, unit, and organization), underscoring their theoretical foundations based on differing composition frameworks. Detailed considerations of composition models is available elsewhere (e.g., Chan, 1998; Kozlowski and Klein, 2000); however, we note here that research on emergent states (e.g., group potency) requires that a level of consensus (i.e., agreement or sharedness), which is based on a theoretically appropriate composition model, be demonstrated. Emergent state research has generally relied on *r_wg_*, intraclass correlations (ICCs), and other agreement statistics (see LeBreton and Senter, 2008, for a review) as indices of consensus. Kozlowski et al. (2013) noted that although these statistical approaches for assessing agreement have been used in both cross-sectional and longitudinal research to demonstrate emergence, their use has predominantly been restricted to static interpretations (even when averaged across time in longitudinal research), and therefore ignores the temporal aspect of emergence. More specifically, in both cross-sectional and longitudinal data, these consensus statistics have been used to demonstrate that emergence has taken place, but only provide a snapshot of sharedness, thereby ignoring the dynamicity of the emergence process. For example, in cross-sectional research, after demonstrating some level of consensus, researchers are left to *assume* a team-level phenomenon has emerged, without actually assessing the pattern of *change in consensus* that may more accurately represent the emergence process (O’Neill and Allen, 2012; Allen and O’Neill, 2015a). Although this is informative from a descriptive standpoint, interpreting isolated ICC estimates may not provide a strict test of whether emergence has occurred. To address this issue, Lang et al. (2018) introduced the consensus emergence model, which allows researchers to examine change in consensus over time, a key component of the emergence process. The current investigation used this methodology to provide an assessment of group potency emergence over time.

As a collective phenomenon, group potency fits into Chan (1998) referent-shift consensus model. Group potency, therefore, requires consensus amongst group members to demonstrate the collective or shared aspect of the construct. Commensurate with Kozlowski et al. (2013) theorizing on emergent processes, group members need time to interact with each other and engage with the task to develop a shared understanding of the team-level phenomenon. Initially, group members’ perceptions of their potency will be based on minimal information as they have had limited time interacting. As a result, initial ratings of group potency will be more indicative of individual members’ perceptions rather than shared perceptions. It can therefore be theorized that agreement between group members will increase over time. Accordingly, we forward the following hypothesis:

Hypothesis 4: Consensus on group potency will increase over time.

## Antecedents of Group Potency’s Dynamic Nature

According to the IPO framework, inputs play an important role in the development of team processes. Inputs are conditions or characteristics of team members that exist prior to the team interacting and performing together, including – but not limited to – personality, and other dispositional characteristics. Inputs can therefore be considered as antecedents to emergent states, such as group potency. We selected conscientiousness and extraversion as two input variables (i.e., resources) that will contribute to group potency (i.e., a resource gain). Our rationale for selecting conscientiousness and extraversion is two fold. First, meta-analytic research by Ng and Feldman (2014), structured around COR theory, demonstrated that both conscientiousness, and extraversion contribute to resource gains (e.g., salary attainment). Second, meta-analytic research by Bell (2007) found that team-level conscientiousness and extraversion were positively related to team effectiveness (ρ = 0.14 and ρ = 0.10, respectively). Although the latter supports the direct relation between our selected inputs and team effectiveness, there is a dearth of research investigating the full IPO framework and the implied indirect effects of *how* the inherently dynamic nature of team processes and resources (e.g., group potency) transmit the effects of input resources to outputs. LePine et al. (2011) described the issues involved with this piecemeal approach of only assessing the input-output, or process-output relations, for example, rather than a more theoretically aligned model of input → process → output. Further, LePine et al. (2011) noted that more advanced research designs and analyses should be forwarded to improve understanding of the complete framework (see also Pitariu and Ployhart, 2010). Finally, Mathieu et al. (2014) pointed out that team personality composition might not just be relevant for static teamwork variables but also their change over time.

In the current research, we investigated the full IPO framework by incorporating team-level conscientiousness and extraversion as inputs (i.e., antecedent resources), initial levels and change in group potency as process variables (i.e., team process resources), and team effectiveness as an output. Together, indirect relations are described with group potency’s dynamics mediating the relations between team-level personality and team effectiveness.

### Conscientiousness

Individuals with high conscientiousness are characterized by being hardworking and achievement-oriented (Goldberg, 1990). Further, conscientious individuals tend to be confident (Chen et al., 2004; Ebstrup et al., 2011), and likely behave in a manner that is conducive to operating in a team environment (e.g., O’Neill and Allen, 2011). Even further, as noted, Bell’s (2007) meta-analysis found that team-level mean conscientiousness was positively related to team performance. Thus, past research has illustrated positive relations between team-level conscientiousness and both group potency and team effectiveness.

We again draw upon COR theory, and apply a resource-based perspective to propose how team-level conscientiousness relates to the dynamics of group potency and team effectiveness. Another key proposition of COR is that initial resources can combine to positively influence the achievement of desired outcomes, and can help produce gains in resources, or alternatively, can provide additional resources to help maintain resources levels that may otherwise become depleted over time. Hobfoll (2011) argued that resources should be considered as “caravans,” in which the combined functioning of resources best facilitates achieving desired outcomes (e.g., meeting goals, coping with stress). Based on the importance of team-level conscientiousness, we argue that team-level conscientiousness can function as a team “input” resource that can lead to gains in (i.e., higher) initial group potency. For instance, groups that see themselves as more collectively hard working will likely see themselves as having higher initial confidence in their ability to achieve the team’s goals, because they know they will persist even when the task difficulty increases. In addition, increased team-level conscientiousness may provide another resource to the team to protect against loss of potency resources over time. Thus, teams with higher levels of conscientiousness will be able to better conserve their potency resources over time. This, in turn, will lead to increased team effectiveness. Thus:

Hypothesis 5a: The initial level of group potency will mediate the relation between conscientiousness and team effectiveness.
Hypothesis 5b: The rate of change of group potency will mediate the relation between conscientiousness and team effectiveness.

### Extraversion

Highly extraverted individuals tend to be talkative and sociable (Goldberg, 1990). Research on team-level extraversion has generally revealed positive relations with team performance (Bell, 2007), as it may facilitate positive interpersonal interactions between team members (Barry and Stewart, 1997). Further, extraverts tend to have higher confidence in their ability to work in a self-managed group (Thoms et al., 1996), suggesting a positive relation between team-level extraversion and group potency. Finally, extraversion involves facets related to energy, activity, and excitement seeking (Hastings and O’Neill, 2009), all of which would encourage strong willingness to engage in the work and exploration required for team success.

Similar to team-level conscientiousness, team-level extraversion can be considered a resource that is brought to the team by its individual members and functions as an input for team processes (i.e., group potency). Thus, considering team-level extraversion as a team resource, it may lead to increased initial group potency and help teams preserve their group potency over time. This will permit teams to conserve and maintain their potency resources during its lifecycle, potentially leading to increased team effectiveness. Based on this theorizing, the following is hypothesized:

Hypothesis 6a: The initial level of group potency will mediate the relation between extraversion and team effectiveness.
Hypothesis 6b: The rate of change of group potency will mediate the relation between extraversion and team effectiveness.

## Materials and Methods

### Participants and Procedure

This study was reviewed and approved by Western University’s Non-Medical Research Ethics Board and participants provided written informed consent prior to participating. Participants were 337 first-year engineering students. The majority of participants (81%) were male, and ranged in age from 16 to 33 years (*M* = 18.5, *SD* = 1.9). Participants were randomly assigned to one of 77 project teams, which consisted of either four (62% of teams) or five (38%) members. Each team had two small design projects (taking place over 2 months each) and one large design project (taking place over 4 months) to complete over the course of an academic year. For the large design project, students were required to create a prototype of a device that individuals with a disability could use to improve their well-being.

Survey data were collected at five different time points throughout the academic year. Conscientiousness and extraversion data was collected on the first day of class before students were assigned into their project teams (i.e., Time 1). Group potency data was collected at three subsequent time points: 2 months (Time 2), 5 months (Time 3), and 8 months (Time 4) after the start of the semester. Grades on the large design project were collected at the conclusion of the semester (i.e., Time 5) and serve as our measure of team effectiveness.

### Measures

#### Conscientiousness

Conscientiousness was measured with ten items from the International Personality Item Pool (IPIP; Goldberg et al., 2006; α = 0.81). The IPIP items correlate highly with Costa and McCrae’s (1992) NEO-PI-R. There were five positively worded and five negatively worded items. A sample item is “I am always prepared.” Participants responded to these items on a five-point Likert-type agreement scale (1, strongly disagree; 5, strongly agree).

#### Extraversion

Extraversion was also measured with ten items from the IPIP (Goldberg et al., 2006; α = 0.86) that correlate highly with the NEO-PI-R. There were five positively worded and five negatively worded items. A sample item is “I feel comfortable around people.” Participants responded to these items on a five-point Likert-type agreement scale (1, strongly disagree; 5, strongly agree).

#### Group Potency

Group potency was measured with seven items from Guzzo et al. (1993), which measure a team’s confidence in their general ability to be effective. A sample item is “No task is too tough for this team.” Participants responded to these items on a five-point Likert-type agreement scale (1, strongly disagree; 5, strongly agree). Sosik et al. (1997) found that these group potency items have strong internal consistency with a Cronbach’s α ranging from 0.87 to 0.98 across three time points.

#### Team Effectiveness

Associated with the large design project, teams submitted a comprehensive written report that was typically about 100 pages in length. The report contained a variety of detailed information pertaining to the project including, design sketches, mathematical models, and implications for practice. Team reports were rated based on their overall quality by experienced course instructors, who were blind to this study’s objectives, and grades were assigned to the team as a whole (i.e., no unique grades were assigned to individual members). Each rater rated a unique subset of the reports (see O’Neill et al., 2018).

#### Analytical Procedure

**FIGURE 1:**
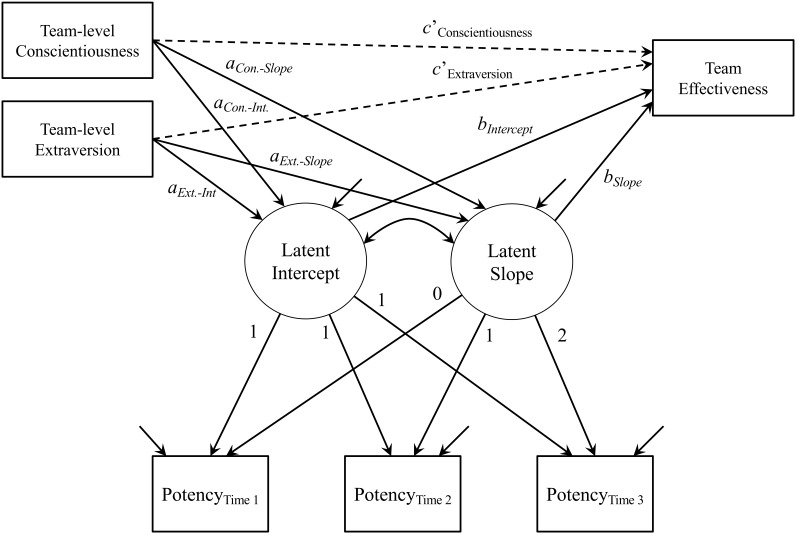
Focal analytical model. Numeric factor loadings for LGM presented. Direct effects, c’paths, and shown in dashed lines. Indirect effects, comprising respective *a* and *b* paths and associated a_*j*_ × b_*j*_ effects, and shown in solid lines.

Using M*plus* 7.4 (Muthén and Muthén, 2012, 2015) throughout for our focal analyses, we implemented a sequential model testing procedure to conduct (1) longitudinal measurement invariance analyses, (2) latent growth modeling, and (3) consensus emergence modeling (Lang et al., 2018). The full model assessed is illustrated in Figure 1. Examinations of change over time requires measurement invariance to ensure that a measure functions and means the same thing over time, and to facilitate meaningful longitudinal inferences (Ployhart and Vandenberg, 2010). Longitudinal measurement invariance assesses the stability of a scale’s measurement model over time, and without this support misleading interpretations may result, akin to comparing apples to oranges over time (Chen and West, 2008). Demonstrating invariance requires several analytical steps, which include: (a) configural invariance, (b) metric invariance, (c) scalar invariance, and (d) strict invariance. Ployhart and Vandenberg (2010) noted that configural, metric, and scalar invariance are sufficient for longitudinal invariance, yet strict invariance was also investigated as it can provide additional insight into the structure and function of a scale (McLarnon and Carswell, 2013). The configural invariance model assesses whether the same pattern of factor loadings holds over time. For determining configural invariance, we – in part – assumed support because all seven potency items, which measure a single factor, were assessed at each time point. In addition, we also considered indicators of model-data fit rendered by the comparative fit index (CFI) and root mean square error of approximation (RMSEA). CFI values > 0.95 and RMSEA values <0.08 can be taken as evidence for acceptable model fit (e.g., Hu and Bentler, 1999). Building on the configural invariance model, metric invariance then constrains respective factor loadings to equality, scalar invariance places additional equality constraints on respective intercepts, and strict invariance places equality constraints on respective item residuals. To assess plausibility of each of these sets of invariance constraints, the Δχ^2^ test can be used because each set of constraints imposed represent a nested model. However, as Δχ^2^ may be overly sensitive to sample size, changes in the CFI of less than 0.010 and/or changes in the RMSEA of less than 0.015 can support invariance in each step (Chen, 2007). In each longitudinal invariance analysis, autocorrelated residuals were specified between respective items (Little, 2013).

Our invariance analyses used individual-level data in order to achieve a balance between sample size and model complexity. However, to account for the nested nature of our data (i.e., individuals within teams), we used robust maximum likelihood estimation, implemented as M*plus*’ MLR estimator, in conjunction with the TYPE = COMPLEX specification to furnish model fit indices and standard errors that were robust to non-independence (Muthén and Muthén, 2012; McNeish et al., 2017). Given the use of the MLR estimator, Δχ^2^ nested model comparisons were facilitated through Satorra and Bentler’s (2001) scaled Δχ^2^ statistic.

An additional wrinkle in estimating the longitudinal invariance models concerns the correct specification of the longitudinal null model (Little, 2013), which is used in the derivation of the CFI. If the null model is incorrect, the CFIs used to judge invariance may also be biased and may result in erroneous inferences. As discussed by Widaman and Thompson (2003), the correct longitudinal null model should specify zero covariances between any indicators (as in the typical null model), but equal variances and equal means for respective indicators across time points. As such, our use of the CFI was based on the corrected longitudinal null model.

Then, using latent growth modeling (Chan, 2002), and the aggregated potency scores, we examined the dynamics involved with group potency. First, we estimated an unconditional model to estimate the mean and variability around the latent intercept and slope of group potency. The latent growth model was specified in a typical fashion with the factor loadings for the latent intercepts all fixed at 1.00, and the factor loadings for the latent slope were fixed at zero, 1.00, and 2.00, for each of the measures (i.e., Time 2, 3, and 4; see above), respectively. The parameterization for the slope follows from equal time spacing between Times 2 and 3, and Times 3 and 4, as both reflected 3-month time lags. We then incorporated team effectiveness, as a simultaneous outcome of both the latent intercept and slope, and the personality predictors to assess the indirect effects. Using bias-corrected bootstrapping, with 10,000 samples, indirect effects were deemed significant if their 95% confidence intervals (CIs) excluded zero. Notably, the personality predictors used the mean-aggregation of scores from each individual member and as mean-aggregated personality is not a shared-unit property of a team (Kozlowski and Klein, 2000) justifying aggregation (via ICCs, etc) is therefore not required (e.g., O’Neill and Allen, 2011).

Finally, we used Lang et al.’s (2018) multilevel procedure to examine consensus emergence of group potency. This allowed us to assess emergence of the group-level potency construct from the sharedness, or more specifically the increasing degree of sharedness, of individual members’ ratings over time.

## Results

Table 1 presents the team-level correlation matrix, the intraclass correlation estimates [ICC(1) and ICC(2)] for group potency at each time point, and Cronbach’s alpha internal consistency estimates. Notably, the ICC estimates increased slightly over time, indicating a growing proportion of variance in group potency that could be attributed to the team-level rather than the individual-level. This suggests increasing consensus in perceptions of team potency over time and stronger emergence. We revisit this pattern to more formally substantiate the emergence of group potency and provide a test of Hypothesis 4.

**Table 1:** Team-level Descriptives and Intercorrelations.

	*M*	*SD*	*ICC(1)*	*ICC(2)*	*1*	*2*	*3*	*4*	*5*	*6*
*1*. Conscientiousness	3.66	0.26	–	–	(0.73)					
*2*. Extraversion	3.49	0.26	–	–	−0.00	(0.77)				
*3*. Potency, Time 1	4.06	0.37	0.27	0.60	0.13	0.22	(0.90)			
*4*. Potency, Time 2	4.06	0.52	0.35	0.65	0.24^∗^	−0.06	0.41^∗∗^	(0.93)		
*5*. Potency, Time 3	3.97	0.62	0.37	0.67	0.30^∗∗^	0.00	0.30^∗∗^	0.62^∗∗^	(0.94)	
*6*. Team effectiveness	82.28	11.01	–	–	0.14	−0.16	0.14	0.35^∗∗^	0.32^∗∗^	–

*n = 77. ICCs not applicable to conscientiousness, extraversion, or team effectiveness measures. Individual-level Cronbach’s α estimates given in parentheses on diagonal; Team Effectiveness was a single score (grade), therefore reliability could not be estimated. ^∗∗^p < 0.01, ^∗^p < 0.05.*

Table 2 presents the results of the longitudinal measurement invariance analyses. The configural invariance model demonstrated adequate fit, CFI = 0.95 and RMSEA = 0.06. Adding equality constraints on the factor loadings resulted in ΔCFI = –0.001 and ΔRMSEA = –0.002, supporting metric invariance. This suggests that the potency measure retains a similar meaning across occasions. The scalar invariance model resulted in a ΔCFI = –0.003 and ΔRMSEA < 0.0004 versus the metric invariance model. This lends support to scalar invariance, which suggests that the potency measure functions similarly over time. As a final stage in the invariance analyses, additional equality constraints were placed on respective item residuals to assess strict invariance. This model resulted in ΔCFI = 0.003 and ΔRMSEA = –0.004, supporting strict invariance, and suggests that each item had equivalent reliability over time. Together, these invariance analyses suggest equivalence of group potency over time, facilitating our focal latent growth models.

**Table 2:** Longitudinal measurement invariance analyses.

	χ^2^	χ^2^c	*df*	#*fp*	CFI	RMSEA	Δχ^2^	Δχ^2^ *df*	ΔCFI	ΔRMSEA
Configural	355.46^∗^	1.22	165	87	0.95	0.06	–	–	–	–
Metric	370.09^∗^	1.21	177	75	0.95	0.06	13.61	12	−0.001	−0.002
Scalar	392.72^∗^	1.20	189	63	0.95	0.06	21.12^∗^	12	−0.003	+0.001
Strict	394.32^∗^	1.27	203	49	0.95	0.06	13.75	14	0.003	−0.004

*χ^2^c, scaling correction factor for χ^2^; df, degrees of freedom; #fp, number of parameters estimated; CFI, comparative fit index (calculated using corrected longitudinal null model; Widaman and Thompson, 2003); RMSEA, root mean square error of approximation; Δχ, Satorra-Bentler scaled χ^2^ difference statistic (Satorra and Bentler, 2001); Δχ^2^ df, degrees of freedom for Satorra-Bentler Δχ^2^; ΔCFI, ΔRMSEA, change in CFI and RMSEA estimates, respectively, from less restricted to more restricted models (i.e., change in CFI from configural invariance model to metric invariance model). ^∗^p < 0.05.*

Given the ICCs provided support for aggregating group potency to the team-level, we averaged individual members’ group potency scores within each team, and used the aggregated scores to estimate our latent growth model. The unconditional growth model demonstrated adequate fit to the data, χ^2^(1) = 0.73, *p* = 0.39, CFI = 1.00, RMSEA = 0.00. With respect to Hypothesis 1, the mean of the latent slope was of central interest, which was estimated as −0.07, *p* < 0.05. This supported Hypothesis 1, suggesting that group potency *decreased* over time (by 0.07 units at each time point). The estimate of the latent intercept was 4.06, *p* < 0.01, and the variances for the latent intercept and slope were 0.20, *p* < 0.01, and 0.04, *p* < 0.05, respectively. The correlation between the latent intercept and slope was −0.14, *p* = 0.59. Interestingly, freeing the slope’s factor loading for the second group potency measure, as in a latent basis model (Grimm et al., 2013) did not suggest an improvement in fit. Specifically, Δχ^2^(1) = 0.71, *p* = 0.39, and both the Akaike Information Criteria and Bayesian Information Criteria were higher in the latent basis model than the latent growth model. Thus, based on parsimony, we proceed with the linear latent growth model. Notably, even in the latent basis model, the trend did not deviate significantly from a linear trajectory, thus lending further credibility to Hypothesis 1, and the underlying linear, downward pattern of change in group potency. Next, incorporating team effectiveness as a simultaneous outcome of the latent intercept and slope factors also resulted in adequate model-data fit: χ^2^ (2) = 1.22, *p* = 0.54, CFI = 1.00, RMSEA = 0.00. Specifying regressions between both intercept and slope factors and effectiveness revealed that the regression of effectiveness on the latent slope was *b* = 0.07, *p* = 0.99, but that for the latent intercept it was *b* = 10.23, *p* < 0.01. Thus, there was no influence of change in potency on team effectiveness, but the starting point of teams’ potency was positively related to effectiveness. Accordingly, Hypothesis 2 was not supported, whereas Hypothesis 3 was supported.

To more formally assess the emergence of the group potency construct, we used Lang et al. (2018) consensus emergence model. This model uses longitudinal changes in the individual-level residual variances as evidence of emerging consensus. Specifically, decreasing residual variances can be taken as indicative of increasing consensus emergence, and therefore reflects more agreement about a team-level phenomenon. Indeed, in our model the estimated change in residual variance was δ = –0.11, *p* < 0.05. This suggests significantly less individual-level variance and comparably greater sharedness at the team-level over time. In other words, this negative coefficient supports the proposition that group potency demonstrated significant increases in the support for emergence over the three measurement occasions. Thus, Hypothesis 4 was supported.

Finally, we incorporated the conscientiousness and extraversion team-level predictors into the latent growth model. This also resulted acceptable model-data fit: χ^2^ (4) = 3.02, *p* = 0.55, CFI = 1.00, RMSEA = 0.00. Neither of the indirect effects involving the latent slope had 95% CIs that excluded zero: the conscientiousness → latent slope → team effectiveness indirect effect was −0.01, 95% CI = –3.11–2.65, and the extraversion → latent slope → team effectiveness indirect effect was 0.08, 95% CI = –2.36–4.55. The indirect effect involving extraversion → latent intercept → team effectiveness was also not significant, −0.73, 95% CI = –9.34–4.65. However, the indirect effect of conscientiousness → latent intercept → effectiveness was significant, 5.57, 95% CI = 0.59–23.58. Thus, there was no evidence for the mediating role for change in potency, but instead the latent intercept transmitted the effect of conscientiousness on team effectiveness. In sum, Hypothesis 5a was supported, but Hypotheses 5b, 6a, and 6b did not receive support.

## Discussion

There are four intriguing findings from the current investigation that contribute to both the group potency and the multilevel emergence literatures. First, the latent growth model revealed a significant negative slope for group potency. Group potency levels therefore *decreased* over time, on average across teams. Previous research by Lester et al. (2002) also found a decrease in group potency over time; however, that study had only two time points and a much shorter time span in comparison to the current investigation (i.e., 9 weeks vs. 6 months, respectively). We theorized that individuals would generally tend to start with high expectations of how their team would perform (Svenson, 1981). As well, due to the “better-than-average” effect when teams first get together they may experience a “honeymoon period” where they have unrealistic positive expectations of how they will do as a group (Forsyth, 2018). Over time, it is probable that the honeymoon dissolves as team members spend more time interacting, debating, dealing with internal conflicts, and other challenges associated with teamwork and the team task (O’Neill and McLarnon, 2018; O’Neill et al., 2018). In this study, we drew upon COR theory to argue that these challenges negatively affect team resources (e.g., group potency), resulting in a decrease in magnitude over time. Interestingly, similar results have been found in other research domains. For example, in examining changes in organizational commitment, an integral workplace resource, Lance et al. (2000) and Bentein and Meyer (2004) found that organizational newcomers experienced loss of this resource over time as they interacted with their new settings. Our results, and those from the domain of organizational commitment, therefore support the argument that resources can be depleted over time as individuals interact with their environment, whether the environmental context is a workplace or a team. This suggests that early team experiences (i.e., socialization) are important for establishing strong, initial group potency resources.

This paved the way for the second intriguing finding from this study: in the latent growth model, teams’ initial group potency predicted overall team effectiveness. This implies that, although group potency takes time to emerge (which we discuss subsequently), early interactions might play an important role in setting a team up for future success. Although teams may have elevated potency ratings during a honeymoon period, they are still able to effectively leverage their potency resources, such that it helps explain teams’ effectiveness later on during project completion (i.e., 6 months later). This finding supports Kozlowski et al. (2013) argument that it is important to assess emergent states as early in a team’s lifecycle as possible. Even though group potency resources may decrease over time, early potency, and the intrateam resources it provides, may have a role in determining future strategizing, planning, and cooperation, which helps to set the stage for the future goal and task accomplishment. Thus, despite the decreasing trend experienced by teams over time, what appears to be an important component of a team’s effectiveness is each team’s perception of potency early on in their respective lifecycle.

The third intriguing contribution that this research provides is that we documented an increase in consensus on group potency within teams. Thus, members gained an increasingly *shared* perception of their group’s potency over time. This is an important aspect of what Kozlowski et al. (2013) described generally as exemplifying the multilevel emergence process: as team members interact they will develop a stronger, shared understanding of the team’s emergent properties (e.g., group potency). Historically, “sharedness” or consensus has only been investigated using cross-sectional analyses, and inferred via ICC estimates, with “high values” taken to support the occurrence of emergence. This approach, however, does not facilitate an inference of the actual process of consensus emergence, which is temporally defined. Using Lang et al.’s (2018) methodology, we were able to utilize an analytical approach that is sensitive to emergence’s inherently temporal nature and provide an empirical estimate of group potency’s emergence. Commensurate with Allen and O’Neill (2015b), we found support for early emergence, with Time 1 ICCs meeting acceptable levels of agreement (LeBreton and Senter, 2008). Nevertheless, our findings also suggest that agreement still increased over longer durations as team members interact and get a better understanding of “who they are” as a collective.

Although the findings of *decreasing* group potency levels and *increasing* consensus on group potency may seem in opposition, these are independent phenomena. Conceivably, consensus could emerge over any level of a construct, which could be static or dynamic in nature. Future research may be able to leverage Lang et al.’s (2018) framework and incorporate predictors of emergence, such as relationship and process conflict (O’Neill et al., 2018), psychological safety (Edmondson, 1999), intrateam communication, and peer feedback (Donia et al., 2018), among others.

The fourth important finding reflects the application of the IPO framework to test key COR principles. More specifically, two input resources – conscientiousness and extraversion – were included as antecedents of group potency’s dynamic nature. We found that the relation between conscientiousness and team effectiveness was mediated by initial group potency. Contrary to our expectations, no effect was found for extraversion, or for the link between conscientiousness and team effectiveness, as mediated by the rate of change in potency level. These findings suggest that teams that comprise individuals with higher levels of conscientiousness are more likely to get off to a “good start,” and utilize their collective personality composition as a resource to develop higher levels of initial group potency (another resource), thereby leading to greater team effectiveness.

### Practical Implications

Stemming from these results, an important practical implication is that early team interactions need to be managed effectively to enable a strong starting point for teams’ group potency. With an emphasis on early group potency, rather than the change in potency over time, teams may be able to leverage initial potency as a critical team resource and more effectively navigate hurdles encountered during project completion. Nevertheless, future research may want to also consider how the potentially negative effects of overconfidence (Goncalo et al., 2010) can be mitigated with early team experiences such as developing a team charter, engaging in informal socialization, and other activities that may assist in developing a healthy level of early group potency.

A second practical implication is that interteam differences in personality composition play an important role in developing early group potency. We found that teams that had members with higher conscientiousness were more likely to develop group potency early on, leading to increased team effectiveness. Drawing from an integration of COR theory and the IPO framework, conscientiousness is an important resource that sets the stage for teams’ early potency, which reflects another critical team resource that, in turn, influences effectiveness. Therefore, teams can utilize the resources made available by their aggregated level of conscientiousness to establish and develop group potency allowing them to be more effective. Thus, it is important to consider personality traits, like conscientiousness, when selecting members for a team (see Allen and West, 2005; Morgeson et al., 2005; O’Neill and Allen, 2011; Allen and O’Neill, 2015a).

### Limitations

One of the limitations of the current study is the use of a student sample that, on average, was relatively young (18.5 years old). As well, the participants were predominantly male. It is therefore somewhat difficult to generalize the current findings to more heterogeneous work environments. Furthermore, our results may only apply to time- and project-limited teams. Teams that are tasked with multiple performance cycles may experience a different form of change in potency over time, during the completion of their projects. Future research will be needed to assess the form and function of potency in alternative types of teamwork, which may also facilitate insight into Marks et al.’s (2001) recommendation to investigate multiphasic perspectives on team processes.

A second limitation concerns the ability to apply these results to the dynamic nature that is exemplified by other emergent states. Specifically, the dynamics of potency may vary from the growth inherent with other emergent states (e.g., cohesion). Although potency may emerge after a relatively short duration, and then decline over time, cohesion (i.e., a motivational force that drives teams to stay together) may take longer to emerge as teams take time to decide whether they want to stay together. Thus, future research should be conducted using similar research methods and analytical procedures (i.e., latent growth modeling, paired with Lang et al.’s (2018) consensus emergence model) to investigate the dynamics of other emergent states.

A third limitation is that the measures marking the beginning of the potency growth trajectories were collected 2 months into teams’ lifecycle. This timeframe was selected because members had limited time to interact over the first 2 months, but would have still been representative of teams’ “honeymoon” levels of potency, as they had yet to receive any substantial feedback on their team effectiveness. The results of this study, however, demonstrate that potency had already begun to emerge by the beginning of the trajectory. Future research should measure and examine potency even earlier on in a team’s inception (see Kozlowski et al., 2013).

### Directions for Future Research

Although this research presents several unique and valuable contributions to the literature, there are a number of crucial questions future research should investigate. First and foremost is cross-validation of these findings with a larger number of more heterogeneous teams engaged in alternative projects that take place over longer (or shorter) time periods and lifecycles. Such research endeavors may highlight alternative forms of group potency change over time (i.e., non-linear, discontinuous). However, we would still likely anticipate consensus to emerge and solidify over time, though it may taper off during longer lifecycles. Though we have substantiated a linear, downward trend in group potency over time, it may also be interesting to examine whether distinct types of teams occupy differential trajectories of group potency dynamics. Specifically, leveraging growth mixture modeling, future researchers could examine nuanced trajectories of potency that may be illustrated by distinct *types* of teams (Muthén, 2001; McLarnon and O’Neill, 2018; O’Neill et al., 2018).

Additionally, future research could be dedicated toward whether similar emergent states (e.g., collective efficacy) may exhibit differential patterns of change over time. For instance, collective efficacy, as previously mentioned, is an emergent state that represents a group’s confidence in their ability to perform a *specific* task, rather than the general ability to perform that is measured by group potency. Thus, as teams engaged in a specific task (e.g., a product development initiative), they could experience increasing collective efficacy as they gain task-specific knowledge, and expertise through practice – similar to how training can increase self-efficacy (Blume et al., 2010) – while also experiencing decreasing group potency as they recognize how challenging it can be to effectively function as a team, in general. Nonetheless, we believe the current research provides substantial value to the literature, and our methodological approach may assist future studies, which we eagerly await so as to equip the literature with a comprehensive understanding of form, function, predictors, and implications of group potency.

## Conclusion

The current investigation improves our understanding of the dynamic aspect of group potency. Results demonstrated that potency decreased over time, which we attributed to a honeymoon period associated with a team’s early interactions. Further, teams tended to agree more on their team’s potency over time, suggesting that it takes time for the group potency construct to emerge. Even further, early group potency predicted team effectiveness, however, the change in group potency did not. This suggests that early interactions play an important role in establishing group potency, which may emerge relatively quickly, and may set the tone for future success. Finally, initial group potency mediated the relation between team-level conscientiousness and team effectiveness, suggesting that conscientiousness plays an important role in influencing the dynamics of group potency, which subsequently leads to increased team effectiveness.

## Ethics Statement

This study was carried out following the protocol approved by the university’s Non-Medical Research Ethics Board, which in accordance with the Declaration of Helsinki, all subjects provided written informed consent prior to participating.

## Author Contributions

HW developed the study idea, coordinated the data management, and wrote sections of the manuscript. MM analyzed the data and wrote sections of the manuscript. TO’N developed the study materials, coordinated the data collection, and provided the comments throughout the manuscript.

## Conflict of Interest Statement

The authors declare that the research was conducted in the absence of any commercial or financial relationships that could be construed as a potential conflict of interest.
